# MRI compatible hemodynamic recording system

**DOI:** 10.1186/1532-429X-15-S1-P22

**Published:** 2013-01-30

**Authors:** Bo Xiao, John W Kakareka, Randall H Pursley, Thomas Pohida, Robert J Lederman, Anthony Faranesh

**Affiliations:** 1 Computational Bioscience and Engineering Laboratory, CIT, NIH, Bethesda, MD, USA; 2 Cardiovascular and Pulmonary Branch, NHLBI, NIH, Bethesda, MD, USA

## Background

During cardiovascular interventions it is critical to have high-fidelity hemodynamic recordings of physiologic signals for patient safety and procedure guidance. MRI compatible monitoring systems are low-fidelity and not suitable for cardiovascular interventions. We present an MRI compatible hemodynamic recording system designed for MRI guided cardiovascular interventions. It is capable of recording 12-lead ECG, two invasive blood pressures, and pulse oximetry (SpO2), and interfaces to a commercial hemodynamic recording system (Siemens SENSIS).

## Methods

**Figure 1:**
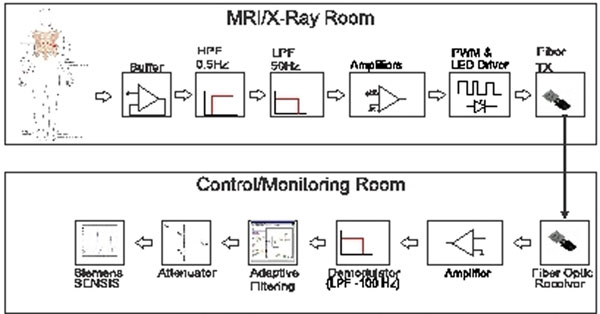
Block diagram of fiber optic based hemodynamic recording interface. Adaptive filtering block (implemented in LabView) is used to remove gradient noise from ECG signals, but is not necessary for invasive pressure or SpO2 signals.

A block diagram of the system is shown in Figure 1. An MRI compatible ECG lead set (InVivo) was attached to the patient. Invasive blood pressure was measured using fluid filled catheters attached to transducers (Edwards TruWave). SpO2 was measured with a commercial sensor attached to an OEM module (Nonin). All signals sensed in the MRI scanner were converted to optical signals and transmitted over fiber optic cables through the waveguide. Adaptive filtering (implemented in LabView) was used to suppress gradient artifacts in the ECG signals. The signals were then converted back to appropriate electrical signals and recorded with SENSIS. It should be noted that any commercial recording system may be used. The system can record a full 12-lead ECG (current prototype connects to 7 electrodes, but is easily expandable), 2 channels of invasive blood pressure and SpO2. Studies on patients and normal volunteers were performed with IRB approval and written consent. MRI guided right heart catheterization was performed on patients using a real-time multi-slice SSFP sequence for image guidance.

## Results

Recordings were made during real-time multi-slice SSFP imaging (flip angle = 40°, TR = 2.9 ms). The RF and gradient artifact was suppressed in the ECG. The pressure and SpO2 signals were free from artifact. Signals were successfully recorded and archived into the commercial patient hemodynamic recording system.

## Conclusions

The MRI compatible hemodynamic recording system was able to obtain high-fidelity signals for ECG, invasive blood pressure and SpO2 during MRI guided catheterization procedures. It maintains a straightforward design, which can be interfaced with any commercial recording system for conventional processing and archiving. This type of system will be a critical component of MRI guided cardiovascular interventions.

## Funding

This work was supported by the Intramural Research Program of the National Heart, Lung, and Blood Institute.

